# Supplementary material for: Truncation of MAT1-2-7 Deregulates Developmental Pathways Associated with Sexual Reproduction in Huntiella omanensis
Source: Microbiol Spectr. 2022 Sep 26;10(5):e01425-22. doi: 10.1128/spectrum.01425-22 (PMC9602353; doi:10.1128/spectrum.01425-22)
Supplement: SUPPLEMENTAL FILE 1 — Supplemental material. Download spectrum.01425-22-s0001.pdf, PDF file, 1.0 MB [file spectrum.01425-22-s0001.pdf]

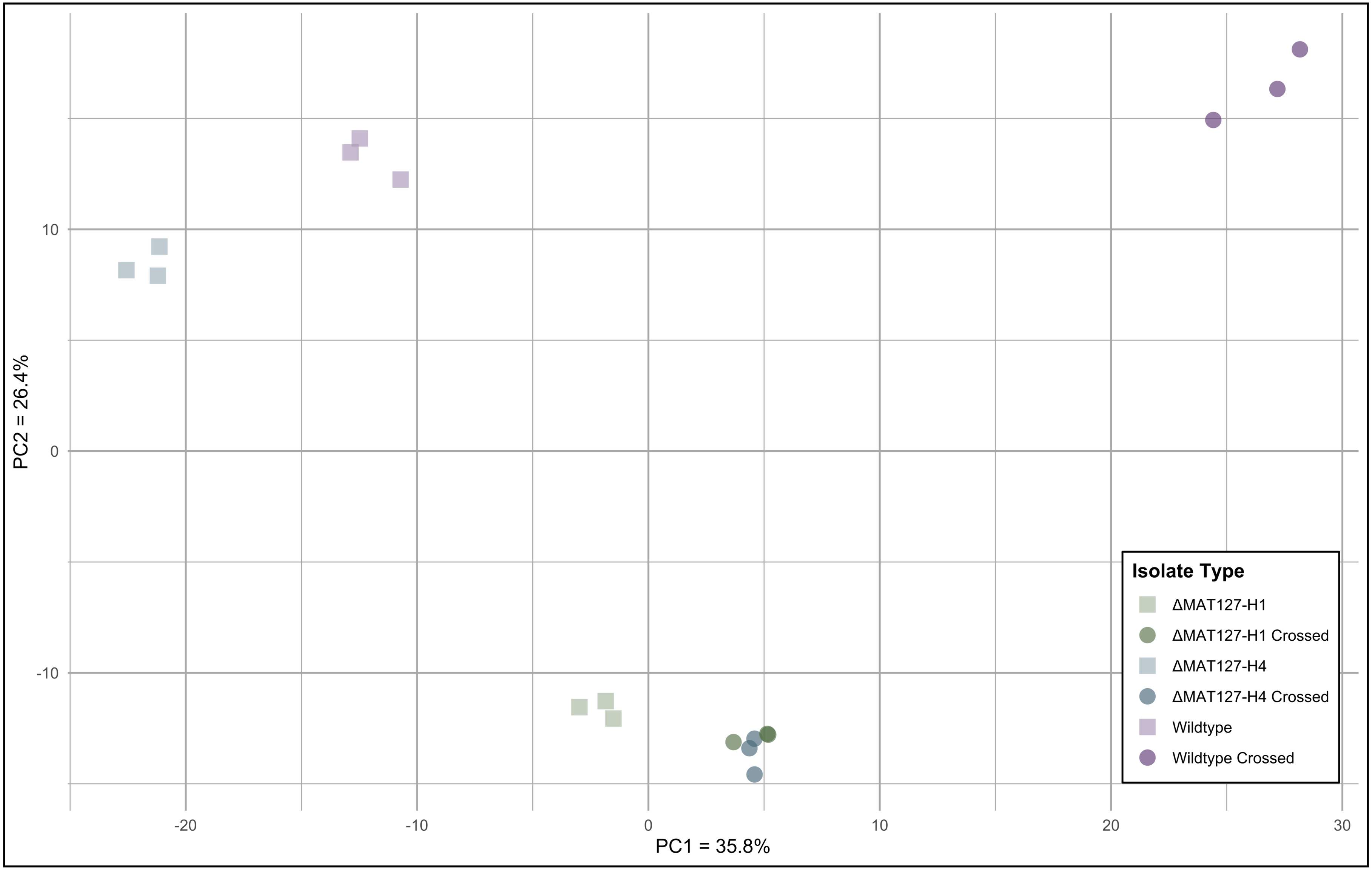

Figure S1: Principal component analysis (PCA) of the technical RNA-seq repeats. The technical repeats of each culture type clustered as expected and a total of 62% of the total variation was explained by the first two components. Square, light-coloured nodes represent the vegetative isolates, while circular, dark-coloured nodes represent the crossed culture types.

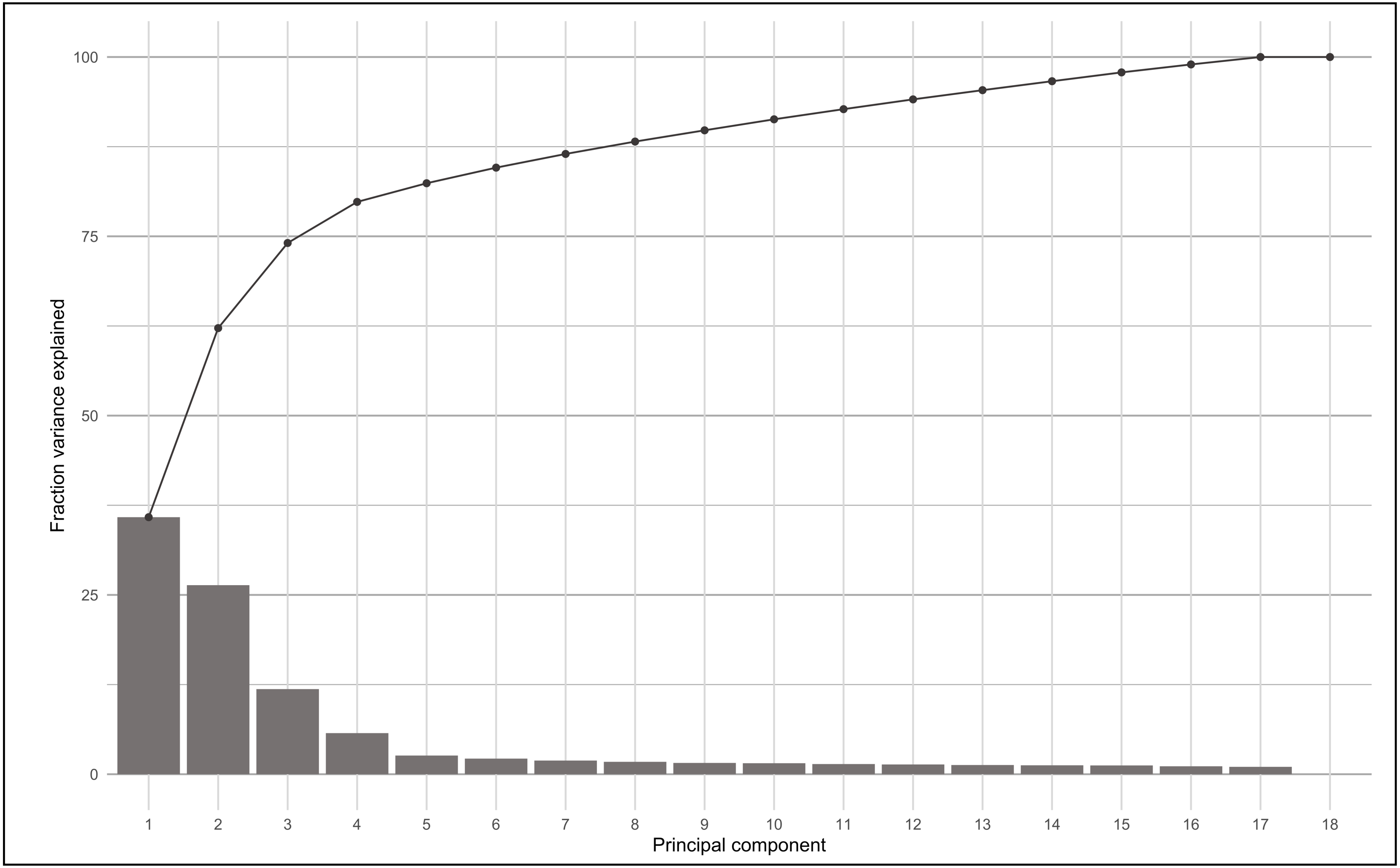

Figure S2. Screen plot from the principal component analysis (PCA). Almost 36% of the variance is explained by the first component and a further 26% is explained by the second component.
